# Comparative Genome-wide Analysis and Expression Profiling of Histone Acetyltransferase (HAT) Gene Family in Response to Hormonal Applications, Metal and Abiotic Stresses in Cotton

**DOI:** 10.3390/ijms20215311

**Published:** 2019-10-25

**Authors:** Muhammad Imran, Sarfraz Shafiq, Muhammad Ansar Farooq, Muhammad Kashif Naeem, Emilie Widemann, Ali Bakhsh, Kevin B. Jensen, Richard R.-C. Wang

**Affiliations:** 1 School of Life Sciences, Tsinghua University, Beijing 100084, China; imran_m1303@yahoo.com; 2 State Key Laboratory of Plant Cell and Chromosome Engineering, Institute of Genetics and Developmental Biology, Chinese Academy of Sciences, Beijing 100101, China; kashifuaar102@hotmail.com; 3 Department of Environmental Sciences, COMSATS University Islamabad, Abbottabad campus, Abbottabad 22060, Pakistan; 4 Institute of Soil & Environmental Sciences, University of Agriculture, Faisalabad 38000, Pakistan; ansar_1264@yahoo.com; 5 Department of Biology, University of Western Ontario, 1151 Richmond St, London, ON N6A5B8 Canada; ewidema4@uwo.ca; 6 Department of Plant breeding and Genetics, Ghazi University, Dera Ghazi Khan 32200, Pakistan; abakhsh@gudgk.edu.pk; 7 Forage & Range Research, United States Department of Agriculture, Agricultural Research Service, Logan, UT 84322, USA; kevin.jensen@usda.gov

**Keywords:** histone acetyltransferases, genome-wide analysis, fiber, abiotic stress expression profiles, cotton

## Abstract

Post-translational modifications are involved in regulating diverse developmental processes. Histone acetyltransferases (HATs) play vital roles in the regulation of chromation structure and activate the gene transcription implicated in various cellular processes. However, HATs in cotton, as well as their regulation in response to developmental and environmental cues, remain unidentified. In this study, 9 HATs were identified from *Gossypium raimondi* and *Gossypium arboretum*, while 18 HATs were identified from *Gossypium hirsutum*. Based on their amino acid sequences, *Gossypium* HATs were divided into three groups: CPB, GNAT, and TAF_II_250. Almost all the HATs within each subgroup share similar gene structure and conserved motifs. *Gossypium* HATs are unevenly distributed on the chromosomes, and duplication analysis suggests that *Gossypium* HATs are under strong purifying selection. Gene expression analysis showed that *Gossypium* HATs were differentially expressed in various vegetative tissues and at different stages of fiber development. Furthermore, all the HATs were differentially regulated in response to various stresses (salt, drought, cold, heavy metal and DNA damage) and hormones (abscisic acid (ABA) and auxin (NAA)). Finally, co-localization of HAT genes with reported quantitative trait loci (QTL) of fiber development were reported. Altogether, these results highlight the functional diversification of HATs in cotton growth and fiber development, as well as in response to different environmental cues. This study enhances our understanding of function of histone acetylation in cotton growth, fiber development, and stress adaptation, which will eventually lead to the long-term improvement of stress tolerance and fiber quality in cotton.

## 1. Introduction

Nucleosomes, the basic unit of chromatin, are composed of 147 bp of DNA wrapped around a histone octamer (two copies of each of H2A, H2B, H3, and H4 histone proteins). Nucleosomes are dynamic in response to developmental and environmental signals, thus altering the DNA accessibility and DNA-template processes to regulate the various processes in plants, including flowering time, root growth, and response to environmental changes [1,2]. Cells use several mechanisms, including post-translational histone modification and DNA methylation, to regulate the gene expression. The N-terminal tails of histone are subjected to various post-translational modifications, including histone acetylation, methylation, etc. [3]. Histone acetylation is carried out by histone acetyltransferases (HATs) in eukaryotes and is associated with transcriptional activation. Histone acetylation can be detected on different lysine residues of histone H3 and H4. For example, in *Arabidopsis*, K9, K14, K18, K23 and K27 of histone H3, and K5, K8, K12, K16, and K20 of H4 are acetylated [4,5]. HATs are well-conserved in yeast, animals, and plants, suggesting the functional conservation of histone acetylation in transcriptional activation. Plant HATs were classified into different subclasses based on their sequence homology to yeast and animal HATs and their mode of action: (1) The Gcn5-related N-acetyltransferase (GNAT)/MYST (Moz, YBF2, Sas2p, Tip) family, (2) CREB-binding Protein (CBP) family, and (3) TBP-associated factor_II_ 250 (TAF_II_250) family [6].

In plants, the genome-wide analysis of HATs has been performed in several species, including *Arabidopsis* [7], rice [8], *Vitis vinifera* [9], and *Citrus sinensis* [10]. HATs have been widely reported to play an important role in various aspects of plant development, including floral development [11,12,13], root growth [14], and gametophyte development [15]. In addition to developmental functions, HATs are also involved in plant adaptation to various environmental fluctuations, such as salt stress [16], cold stress [17], heat stress [18], light signaling [19], abscisic acid (ABA) [20], and other hormone signaling [21]. Therefore, the understanding of HATs functions in field crops may play an important role in sustainable agriculture and food security.

Cotton, as a major source of natural and renewable textile fiber, holds a significant value in the world economy and in daily human life [22]. *Gossypium hirsutum* is a natural allotetraploid (AADD) that arose from the interspecific hybridization between *Gossypium arboretum* (AA) and *Gossypium raimondii* (DD), which occurred approximately 1–2 million years ago [23]. Because allopolyploid cotton produces a superior quality of fiber with high yield compared with their diploid progenitors [24], *G. hirsutum* is widely grown in many parts of the world and contributes to more than 90% of commercial cotton production [25]. On the one hand, world climate is changing quickly, and the abiotic stresses are severely affecting the cotton yield and fiber quality [26,27]. On the other hand, the increasing world population demands the improvement of the cotton yield to meet the requirements of the textile industry. Therefore, identifying the potential genes conferring resistance to different stresses for the molecular breeding of cotton is of the utmost importance [28]. Although the role of HATs has been investigated in the plant development [29,30], response to environmental signals [16,18,31], and hormone signaling [16] in other plants, little is known regarding the function of HATs in cotton. In this scenario, *G. hirsutum*, *G, raimondii*, and *G. arboretum*, having recently available genomic data [32,33,34,35], provide an excellent opportunity to identify the candidate genes involved in fiber development and biotic and abiotic stress tolerance and to expand our understanding of underlying epigenetic mechanisms.

In this study, we identified HAT genes from the whole genome of *G. hirsutum*, *G raimondii*, and *G. arboretum*. The identified HATs were comprehensively analyzed for phylogenetic classifications, gene structures, identification of conserved motifs and domain organization, and the presence of cis-regulatory elements in their promoters. Furthermore, gene expression profiles of *G. hirsutum HATs* were analyzed during the different stages of fiber development and in response to various abiotic stresses. Such a comprehensive analysis of HATs provides a fundamental understanding of their roles in cotton growth and development. Furthermore, this study will be useful for functional genomic studies on the regulations of histone acetylation and will eventually lead to the long-term improvement of stress tolerance in cotton.

## 2. Results

### 2.1. Identification of HATs in Cotton

A systematic blast search was performed to identify the HATs in the genomes of *G. hirsutum*, *G. raimondii* and *G. arboretum* with the query sequence of *Arabidopsis*, and candidate HAT were identified in the cotton genomes. Then, Pfam and InterProScan databases were used to further verify the candidate HAT, and a total of 18 *G. hirsutum* (GhHATs), 9 *G. raimondii* (GrHATs), and 9 *G. arboretum* (GaHATs) were identified (Table S1). The properties of the identified *Gossypium* HATs were analyzed by ExPASy and we found an open reading frame (ORF) length ranging from 1407–6876 bp, which encoded the polypeptides ranging from 468–2291 amino acids with a predicted molecular weight of 53–256 KD. In addition, the theoretical isoelectronic point (PI) values ranged from 0.28–8.84 (Table S1).

### 2.2. Phylogenetic Analysis of the HAT Gene Family

**Figure 1:**
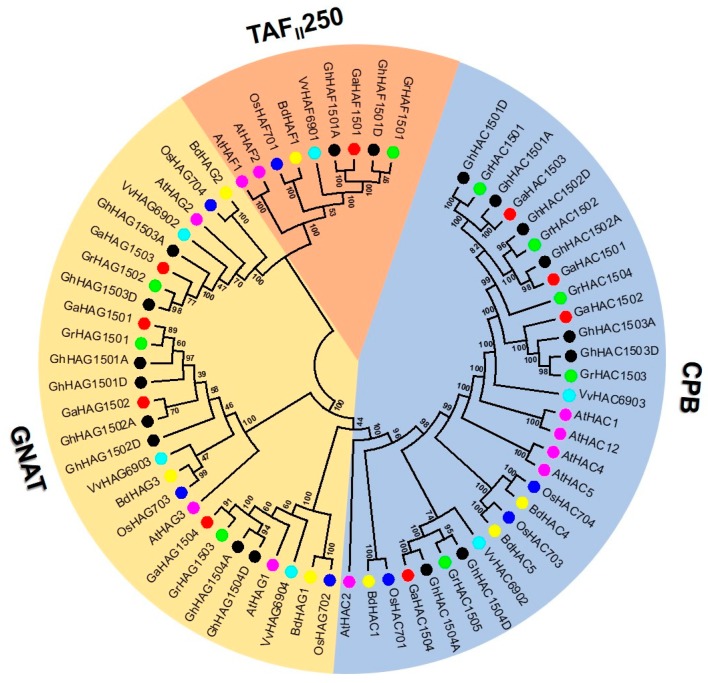
Phylogenetic relationships of histone acetyltransferases (HATs) from *Gossypium hirsutum*, *Gossypium raimondii*, *Gossypium arboretum*, *Arabidopsis thaliana*, *Oryza sativa*, *Brachypodium distachyon* and *Vitis vinifera*. The un-rooted phylogenetic tree was constructed using MEGA 6 by the neighbor-joining (NJ) method, and the bootstrap analysis was performed with 1000 replicates. For the phylogenetic tree, amino acid sequences were used and the classification of CPB, GNAT and TAF_II_250 was performed based on the conserved signature domain of each subgroup. The HATs from *G. hirsutum*, *G. raimondii*, *G. arboretum*, *Arabidopsis*, *V. vinifera*, *Brachypodium distachyon*, and *Oryza sativa* were marked with black, green, red, pink, cyan blue, yellow, and blue dots, respectively.

To investigate the evolutionary relationship of HATs among cotton (*G.raimondii, G. arboretum*, and *G. hirsutum*) and other species, we constructed a phylogenetic tree using MEGA 6.0. We used the newly identified *Gossypium* HATs and previously identified HATs from *Arabidopsis thaliana, Vitis vinifera*, *Oryza sativa*, and *Brachypodium distachyon* to confirm their evolutionary relationship with the un-rooted phylogenetic tree using the neighbor-joining (NJ) method (Figure 1). The phylogenetic tree results showed that similar to *Vitis* and *Arabidopsis*, *Gossypium* HATs could also be grouped into three distinct classes: CPB, GNAT and TAF_II_250. MYST homologs were found absent in the *Gossypium* genomes. To validate the phylogenetic tree constructed using the NJ method, we also used the minimum evolution method to construct a tree and found that HAT genes could be naturally classified into three groups and the members within each clade were stable with little difference between topology, which indicates that the NJ tree method could be used for further analysis.

We found that the number of genes in CPB (30) was greater than that in either GNAT (27) or TAF_II_250 (9). Furthermore, we found that all the three groups comprised mono and dicot species. It is noteworthy that the genes within each group clustered with a dicot- or monocot-specific pattern. The number of HAT from each species was different within each group. Compared to other species, cotton HATs showed a closer relationship with *Vitis* HATs because they always clustered closely to each other in the phylogenetic tree. Nevertheless, their gene numbers were not similar within the group.

### 2.3. Phylogenetic Tree Construction, Conserved Motif and Gene Structure Analysis of Cotton HATs Genes

**Figure 2:**
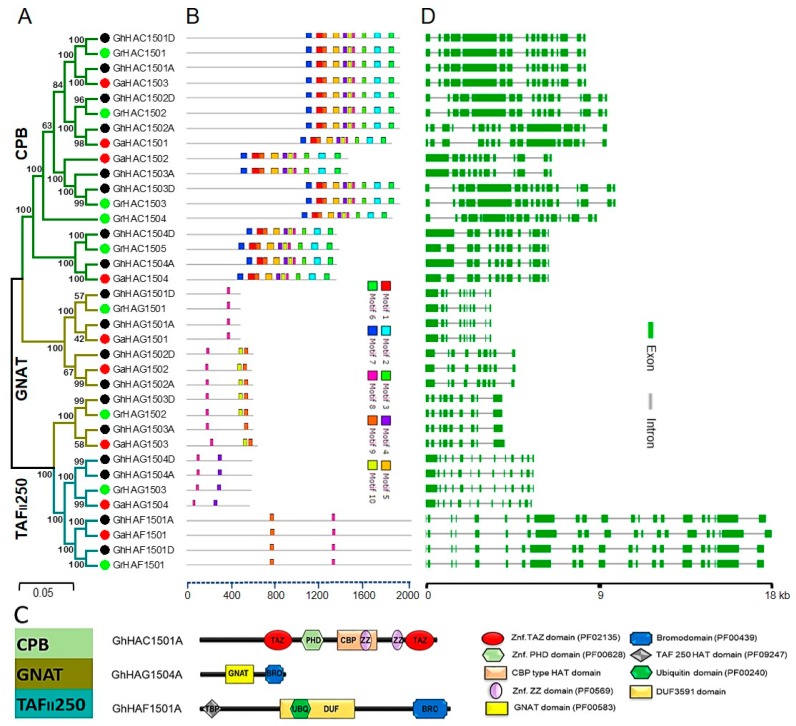
Phylogenetic relationships, conserved motifs, domain organization, and gene structure analysis of HATs in *Gossypium hirsutum*, *Gossypium raimondii* and *Gossypium arboretum*. (**A**) The unrooted phylogenetic tree was constructed using MEGA 6 by the NJ method, and the bootstrap analysis was performed with 1000 replicates. The HAT proteins from *G. hirsutum*, *G. raimondii*, and *G. arboretum* were marked with black, green and red dots, respectively. The branches of each subgroup were indicated in a specific color. (**B**) Motif identification analysis in HAT proteins from *G. hirsutum*, *G. raimondii* and *G. arboretum*. Each motif is shown in unique color, and the regular expression and sequences of the 1–10 motifs are listed in Figure S1. (**C**) Domain organization of HAT proteins from *G. hirsutum*, *G. raimondii* and *G. arboretum*. One representative of each subgroup of HAT from *G. hirsutum* is presented. (**D**) The intron/exon structure of *HAT* genes from *G. hirsutum*, *G. raimondii* and *G. arboretum*. The green boxes and grey lines represent exons and introns, respectively.

To confirm the subgroup classification and to determine the evolutionary relationship between *Gossypium* HATs, we generated another un-rooted phylogenetic tree using the neighbor-joining method (Figure 2A). We observed one subgroup related to CBP (HACs) and TAF_II_250 (HAFs) in *Gossypium*, whereas three subgroups related to GNAT (HAGs) were observed. The CBP and GNAT subgroups contained four members in *G. raimondii* and *G. arboretum*, while TAF_II_250 contained only one member. Furthermore, each diploid progenitor had its own ortholog in allotetraploid.

To gain insight into the evolutionary origin and putative functional diversification, a Multiple Expectation Maximization for Motif Elicitation (MEME) analysis was performed, which identified a total of 10 conserved motifs in *Gossypium* HATs (Figure 2B and Figure S1). All members of the CPB subfamily contained all the conserved motifs, whereas GNAT and TAF_II_250 only contained a few conserved motifs. However, the motif 8 was found conserved in all the *Gossypium* HATs, suggesting that the potential catalytic site of *Gossypium* HATs was conserved. We further dissected the motif analysis of *Gossypium* HATs by investigating their domain organization (Figure 2C). The CBP subfamily of *Gossypium* HATs contained plant homeodomain (PHD), Znf-TAZ, Znf ZZ and a CBP-type HAT domain, while the GNAT subfamily of *Gossypium* HATs contained a conserved GNAT and bromodomain. Moreover, the TAF_II_250 subfamily of *Gossypium* HATs contained a bromodomain, ubiquitin (UBQ), TATA box–binding protein (TBP), and domain of unknown function (DUF) domain. Furthermore, an alignment information was produced to explore the amino acid conservation of the GhHATs domain sequence (Figure S2). The multiple sequence alignment analysis revealed that all GhHATs proteins shared regions of conserved polypeptide sequences, which could be involved in their molecular functions (Figure S2A–C). In general, *Gossypium* HATs contained a domain organization similar to that of their counterparts in other species.

We then analyzed the gene structure of HATs from *G. hirsutum, G raimondii* and *G. arboretum* (Figure 2D). Our results showed that the number and length of intron/exons were different among CPB, GNAT and TAF_II_250 classes. For example, intron/exon numbers and gene length of TAF_II_250 were greater than the CPB and GNAT. Furthermore, the gene structure of *G. raimondii* and *G. arboretum* was extremely similar to their orthologs in the *G. hirsutum*. However, in general, gene structure in terms of intron/exon was greatly similar within the subgroup, which was consistent with the phylogenetic analysis.

### 2.4. Chromosomal Distribution and Duplication Analysis of HATs

**Figure 3:**
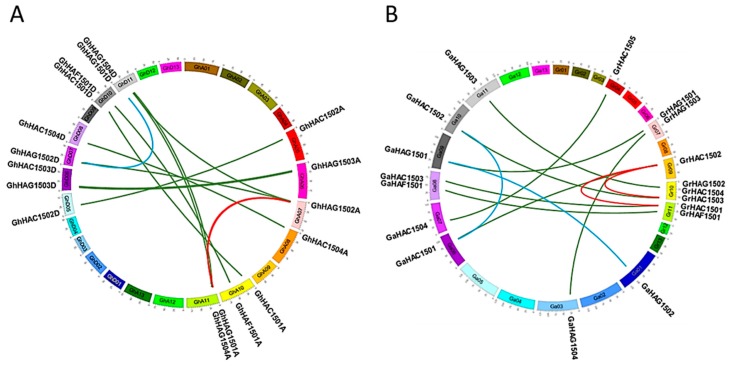
Chromosomal distribution and gene duplication of HATs in *Gossypium hirsutum* (**A**) and in *Gossypium raimondii* and *Gossypium arboretum* (**B**). The chromosome number was indicated in boxes and represented as Gh1-Gh13A/D (**A**), Ga1-Ga13, and Gr1-Gr13 (**B**) for *G. hirsutum*, *G. raimondii*, and *G. arboretum*, respectively. The orthologous HATs are connected by green, while the segment duplication of HATs is represented by light blue and red colors in different genomes.

The *G. hirsutum* HATs were mapped to their corresponding chromosomes (Figure 3A), and all the *HATs* were unevenly distributed on the chromosomes of *G. hirsutum*. For example, all the 18 *G. hirsutum* HATs were assigned to 12 chromosomes out of 26 (Figure 3A). The CBP subfamily of *G. hirsutum* HATs was localized on the chromosomes 5, 6, 8 and 10, while the GNAT subfamily of *G. hirsutum* HATs was localized on the chromosomes 6, 7, 10 and 11. We also mapped the HATs from the diploids *G. raimondii* and *G. arboretum* and found that all the HATs from *G. raimondii* and *G. arboretum* were also unevenly distributed on their chromosomes, similar to *G. hirsutum* (Figure 3B). However, compared with *G. raimondii*, the HATs from *G. arboretum* were more evenly distributed on their chromosomes. We also observed that subfamilies of *Gossypium* HATs were localized to different chromosomes in both diploid progenitors (*G. raimondii* and *G. arboretum*), as well as in the allotetraploid *G. hirsutum*. For example, the TAF_II_250 subfamily member HAF1501 was localized on the chromosome 10 in *G. hirsutum*, and was localized on the chromosome 8 and 11 in *G. arboretum* and *G. raimondii*, respectively.

We also investigated the contribution of gene duplication to the expansion of *Gossypium* HATs (Figure 3A,B). Two segmental duplications were found in *G. hirsutum* (*GhHAC1501A/GhHAC1502A*, *GhHAG1501A/GhHAG1502A*), indicating that duplications occurred in the A genome of *G. hirsutum.* Similarly, two segmental duplications were also found in *G. raimondii* (*GrHAC1501/GrHAC1502*, *GrHAC1502/GrHAC1503*) and in *G. arboretum* (*GaHAC1501/GaHAC1502, GaHAG1501/GaHAG1502*). Furthermore, we also checked the Ka/Ks ratio to explore the selective constraints on each pair of duplicated *Gossypium HAT* (Table S2). The Ka/Ks ratio was found to be less than 0.30 in all the duplicated gene pairs of *Gossypium* HATs, suggesting that all the duplicated gene pairs of *Gossypium* HATs experienced strong purifying selection pressure.

### 2.5. Putative Cis-Elements in the Promoter Regions of GhHATs

To gain more insight into the putative functions of HATs, the putative cis-regulatory elements were scanned in the 1000 bp upstream of the transcription start sites of *G. hirsutum* using the Plant CARE database (Figure S3, Tables S3 and S4). In addition to TATA- and CAAT-box core cis-elements, phytohormone response elements, stress response elements and development response elements were found in the promoters of *G. hirsutum* HATs. Most of the cis-elements were conserved among the CBP, GNAT and TAF_II_250 subfamilies of *G. hirsutum* HATs. However, some cis-elements were absent in some subfamilies. For example, CAT-box (cis-acting regulatory element related to meristem expression), MRE (MYB binding site involved in light responsiveness), P-box (Gibberellin-responsive element), and O2-sites (cis-acting regulatory element involved in zein metabolism regulation) cis-elements were absent in the CBP subfamily, but were found present in GNAT and TAF_II_250 members. Similarly, circadian (cis-acting regulatory element involved in circadian control), skn-1 motifs (cis-regulatory element required for endosperm expression), Box-W1 (fungal elicitor-responsive element), and 5′ UTR Py-rich stretch (a cis-regulatory element conferring high transcription levels) cis-elements were absent in the TAF_II_250 subfamily. Moreover, cis-elements in the promoters of A and D genomes were largely conserved in *G. hirsutum HATs*. However, TGA cis-elements (a cis-acting regulatory element involved in the MeJA responsiveness) were only present in the D genome. We also observed that cis-elements in the promoters of orthologous gene pairs of *G. hirsutum* were largely similar. However, some exceptions were also found where they differed much for the cis-elements, e.g., GhHAC1504-A/GhHAC1504-D and GhHAG1503-A/GhHAG1503-D.

### 2.6. Gene Expression Analysis

#### 2.6.1. Expression Analysis of *GhHATs* in Different Tissues, Developmental Stages and Multiple Abiotic Stresses by RNA-sequencing

**Figure 4:**
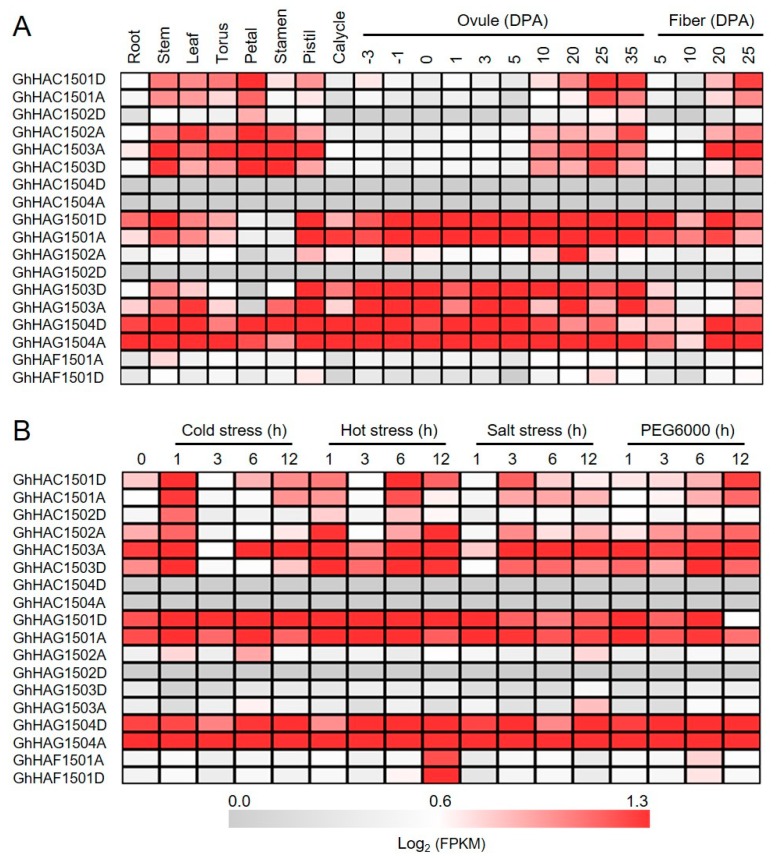
Gene expression pattern of *Gossypium hirsutum HATs* by the analysis of RNA-sequencing in different tissues and at different stages of fiber development (**A**) and in response to cold, hot, salt, and polyethylene glycol (PEG) 6000 stresses (**B**). The illumina reads of RNA-seq data were retrieved from the National Center for Biotechnology Information Sequence Read Archive (NCBI SRA) database. The color scale at the bottom of heat map indicates the fragments per kilobase million (FPKM)-normalized log2 transformed counts.

To better understand the potential physiological functions of GhHATs in allotetraploid cotton, we investigated the expression of *GhHAT* genes. RNA-sequencing data were downloaded from the National Center for Biotechnology Information (NCBI) and analyzed. Their analysis revealed that *GhHATs* were widely expressed in the vegetative (root, stem, and leaf) and reproductive (torus, petal, stamen, pistil, calyx, and −3, −1, 0, 1, 3, 5, 10, 20, 25 and 35 days post-anthesis (DPA) ovule) tissues (Figure 4A), highlighting the diverse biological functions of HATs in different tissues. We also noted that some *GhHATs* did not express in vegetative tissues, but had a very weak expression in the reproductive tissue, e.g., *GhHAG1502-D*. We found that A and D genomes showed preferential expression for only some genes in the leaf, root, and stem. For instance, the expression of *GhHAG1502-A* was higher than *GhHAG1502-D* in all the analyzed tissues. However, the opposite correlation was also observed, e.g., the expression of *GhHAG1504-D* and *GhHAG1503-D* was higher in the root than *GhHAG1504-A* and *GhHAG1503-A*. In addition, we also found that *GhHATs* expression was also differently regulated during the course of development (Figure 4B). For example, *GhHAC1501-D* and *GhHAC1502-A/D* expressions were increased with the development of the root, while the expression of *GhHAC1502-D* decreased with seed cotyledon development. We further investigated the gene expression pattern of *GhHATs* in different abiotic stresses. The expression of *GhHAG1501-A/GhHAG1501-D* and *GhHAG1504-A/GhHAG1504-D* was strongly induced by multiple stresses, indicating their potential involvement in stress responses. However, no clear changes in expression levels were observed for more than half of the *GhHATs* under cold, hot, salt, and polyethylene glycol (PEG) 6000 conditions.

#### 2.6.2. Expression Pattern of *GhHATs* Genes by qPCR

##### Tissue Specific Expression Patterns of HATs

**Figure 5:**
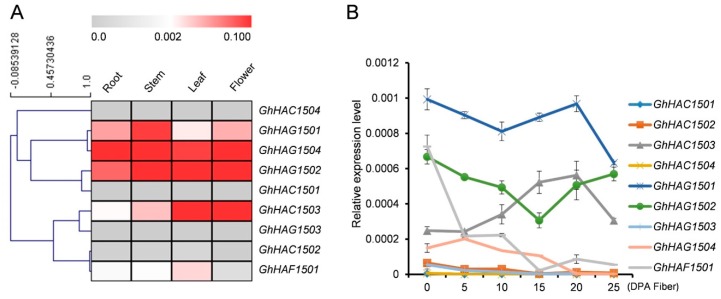
Gene expression validation of *HATs* from *G. hirsutum* by quantitative real-time polymerase chain reaction (qRT-PCR) in different tissues. (**A**) RNA from the root, leaf, stem and flowers was extracted and reverse transcribed. *Ubiquitin 7* (*UBQ7*) was used as an internal control for qRT-PCR. The relative expression is presented in the heat map, and the color scale at the top represents the relative signal intensity. The primer sequences can be found in Table S5. (**B**) Gene expression validation of *HATs* from *G. hirsutum* by qRT-PCR at different stages of fiber development. The data were normalized to *UBQ7*. The data presented are the average of three biological replicates. Bar = standard deviation (SD).

We further validated the *GhHATs* gene expression in some vegetative and reproductive tissues by quantitative real-time polymerase chain reaction (qRT-PCR) (Figure 5A). Because the A and D genomes of allotetraploid cotton were extremely similar in mRNA levels, we considered *GhHAT-A* and *GhHAT-D* as one combination (*GhHAT*) and checked the expression levels by qRT-PCR. Among all the nine analyzed *GhHATs* belonging to three different classes, *GhHAG1501, GhHAG1502, GhHAC1503* and *GhHAG1504* showed the most prominent expression levels in the analyzed tissues, indicating their roles in the development of the leaf, root, stem and flowers. Although *GhHAG1502* and *GhHAC1503* were expressed in the root, stem and leaf, the highest expression was found in leaves. However, only *GhHAG1501, GhHAG1504* and *GhHAG1502* showed the highest expression in flowers, implying the specific function of these *GhHAGs* in flower development. It also indicated the dramatic functional divergence among different classes of GhHATs. Furthermore, *GhHAG1501* and *GhHAG1502* formed a paralogous pair and showed the expression conservation in all the analyzed tissues.

##### Expression of HATs at Different Fiber Development Stages

To explore the potential role of GhHATs in fiber development, we investigated their expression at different developmental stages of fiber development (0–25 DPA) by qRT-PCR (Figure 5B). Among all the *GhHATs*, only *GhHAG1501*, *GhHAG1502*, *GhHAC1503* and *GhHAF1501* showed the prominent expression levels during the different stages of fiber development (Figure 5B), indicating that these four genes may play dominant roles in the fiber development. However, among these four, the expression of *GhHAG1501* and *GhHAG1502* was the highest among the *GhHAGs* subgroup members at 0 DPA, and then gradually decreased during the fiber development (0–25 DPA). Similar to *GhHAGs* family members, the expression of *GhHAF1501* was also decreased during the fiber development, except at 20 DPA, where only the expression of *GhHAF1501, GhHAG1501* and *GhHAG1502* slightly increased and then again decreased at 25 DPA. Contrary to *GhHAF* and *GhHAG* family members, the expression of *GhHAC1503* from *GhHACs* family was gradually increased during the fiber development with a maximum at 20 DPA. Afterward, the expression of *GhHAC1503* was decreased at 25 DPA. We further separated the contribution of A and D genomes for the fiber development of *G. hirsutum* from RNA-seq data analysis and found that the A genome preferentially expressed more than D genome partners in the different fiber developmental stages (Figure 4A).

##### Expression of HATs in Response to Heavy Metals and Abiotic Stresses

**Figure 6:**
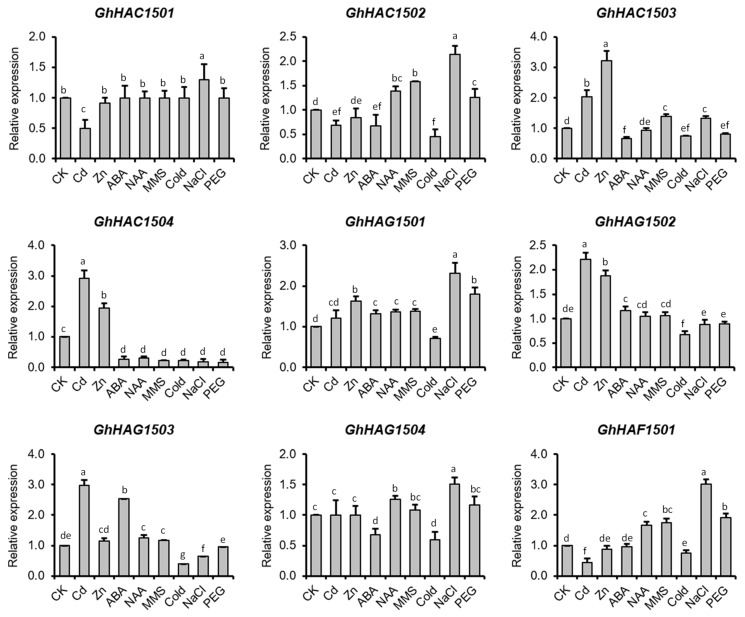
Expression pattern of *Gossypium hirsutum HATs* in response to abiotic stresses and hormones. RNA from roots after 24 h of CK (control), cadmium (Cd), zinc (Zn), abscisic acid (ABA), auxin (NAA), DNA damage (MMS), cold, salt (NaCl), and drought (PEG4000) was extracted and reverse transcribed. *UBQ7* was used as an internal control for qRT-PCR. The data presented are the average of three biological replicates. Different letters indicate significant difference by least significant difference (LSD) test (*p* ≤ 0.05). Bar = SD.

To better understand the role of GhHATs in abiotic stresses, we investigated the gene expression of *GhHATs* in response to metal stress (Cd and Zn), salt stress (NaCl), cold stress and drought stress by qRT-PCR. All the abiotic stresses differentially regulated the expression of *GhHATs* (Figure 6), indicating the functional specificity of GhHATs in response to a particular stress. In response to Cd stress, the expression of *GhHAC1501, GhHAC1502* and *GhHAG1501* decreased, while the expression of *GhHAC1503, GhHAC1504, GhHAG1502* and *GhHAG1503* increased compared with the control. However, the expression of *GhHAG1501* and *GhHAG1504* did not change compared with the control. In response to Zn stress, only the expression levels of *GhHAC1503*, *GhHAC1504*, *GhHAG1501* and *GhHAG1502* increased, while all other studied genes did not show differential expression compared with the control. However, the expression levels of *GhHAC1503*, *GhHAC1504* and *GhHAG1502* were different in response to Cd and Zn stresses despite their increased expression. Furthermore, the expression of *GhHATs* was investigated in response to cold, salt, and drought stresses. The results showed that in response to cold stress, the expression of *GhHAC1502*, *GhHAC1503*, *GhHAC1504, GhHAG1501, GhHAG1502, GhHAG1503, GhHAG1504* and *GhHAF1501* decreased compared with the control. In response to salt stress, the expression of *GhHAC1501, GhHAC1502, GhHAC1503, GhHAG1501, GhHAG1504* and *GhHAF1501* increased, while the expression of *GhHAC1504* and *GhHAG1503* decreased compared with the control. In response to PEG treatment, the expression of *GhHAG1501* and *GhHAF1501* increased, while the expression of *GhHAC1503* and *GhHAC1504* decreased compared with the control.

##### Expression of HATs in Response to MMS

We also investigated the *Gossypium HATs* gene expression in response to the DNA damage agent methyl methanesulfonate (MMS) (Figure 6). The results showed that in response to MMS treatment, the expression of *GhHAC1502, GhHAC1503, GhHAG1501* and *GhHAF1501* increased, while the expression of *GhHAC1504* decreased compared with the control, indicating their potential role in DNA damage repair pathways in cotton.

##### Expression of HATs in Response to Phytohormones

Cis-regulatory elements related to phytohormones were found in the promoter of *GhHATs* (Figure S2, Tables S3 and S4). We therefore investigated the gene expression of *GhHATs* in response to auxin (NAA) and abscisic acid (ABA) by qRT-PCR. Auxin and ABA treatments differentially regulated the expression of *GhHATs* (Figure 6). The results showed that in response to ABA treatment, the expression of *GhHAC1503, GhHAC1504* and *GhHAG1504* decreased, while the expression of *GhHAG1501* and *GhHAG1503* increased compared with the control. In response to NAA treatment, the expression of *GhHAC1502, GhHAG1501* and *GhHAF1501* increased, while the expression of *GhHAC1504* decreased compared with the control.

### 2.7. Co-Localization of HATs with QTLs of Fiber Development

**Figure 7:**
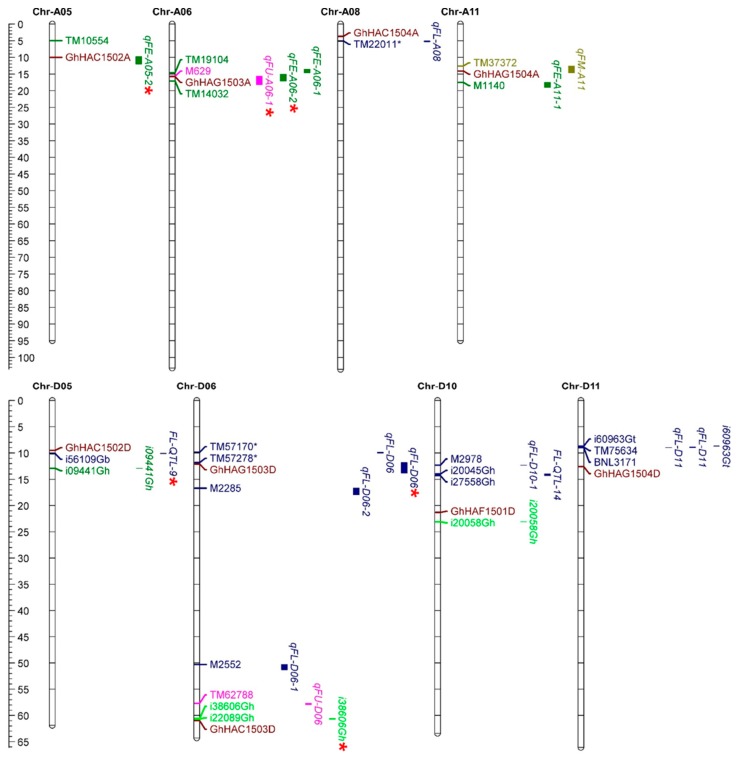
Distribution of co-localized *HAT* genes on chromosomes of A and D subgenomes of *G. hirsutum*. The scale represents the physical position of genes and quantitative trait loci (QTL)-linked markers in megabases (Mb). QTLs/Single Nucleotide Polymorphism (SNPs) related to fiber length (FL), fiber elongation (FE), fiber micronaire (FM), fiber strength (FS), and fiber uniformity (FU) are shown. Asterisks indicate that the *HAT* genes co-localized with QTLs/SNPs related to fiber.

To validate the potential function of GhHATs in fiber development, the co-localization of *GhHATs* with reported QTLs/SNPs of fiber development (i.e., fiber length (FL), fiber elongation (FE), fiber micronaire (FM), fiber strength (FS), and fiber uniformity (FU) was analyzed. Nine genes were mapped to eight chromosomes with reported QTLs of FL, FE, FS, FM, and FU (Figure 7). Among nine *GhHATs*, only four genes were co-localized with QTLs of FL, FE, FM, FS, and FU on four chromosomes, i.e., Chr-A05, Chr-A06, Chr-A08 and Chr-A11 of the A subgenome. *GhHAC1502* was located within the qFE-A05-2, *GhHAG1503* gene anchored in qFU-A06-1 and qFE-A06-2, and *GhHAG1504* was mapped in the qFU-A05-2 QTL region, while *GhHAC1504* was 2 Mb from the qFL-A08. Among the D subgenome *HATs*, *GhHAC1502*, *GhHAC1503* and *GhHAG1503* were anchored in the FL-QTL-9, SNP (i20058Gh, i38606Gh), and qFL-D06, respectively, while *GhHAF1501* and *GhHAG1504* were found in surroundings of reported SNP. Interestingly, some genes were co-localized with multiple QTLs related to different fiber development traits. For example, the *GhHAC1502* gene on chromosome A05 and D05 was anchored in FL and FE QTL, and the *GhHAG1503* gene was localized inside the FL, FE and FU QTL. This reveals that these *GhHATs* had pleiotropic effects on fiber development related traits.

## 3. Discussion

Histone acetylation is a mark of transcriptional activation and has been reported to play an important role in plant development and response to various biotic and abiotic stresses [12,14,15,16,18]. Moreover, levels of histone acetylation are tightly linked with gene expression regulation. HATs carry out histone acetylation and have been classified into different distinct groups in *Arabidopsis* [7], rice [8], *Vitis vinifera* [9], and *Citrus sinensis* [10]. In this study, we revealed that *Gossypium* HATs could also be classified into three major subgroups: CBP, GNAT, and TAF_II_250. This suggests that multiple subgroups of *Gossypium* HATs might play specialized roles in the adaptive evolution of cotton. However, similar to rice [8], the MYST domain containing homologs of HATs in all the three genomes of cotton were absent, while *Arabidopsis* [7] and *Vitis* [9] had MYST domain members. This indicates the specific functional divergence of HATs between the different field crop plants. CBP, GNAT, and MYST HATs are also considered as transcriptional co-activators in addition to their HAT activity. For example, TAZ-type, ZZ-type and PHD-type zinc finger domains of CBP have been reported to play an important role in protein recognition and protein-protein interactions [36,37]. The PHD domain has also been reported to interact with histones and other histone-related proteins [38]. Furthermore, bromodomains are known to bind to acetylated lysine residues [39,40]. Along with functional catalytic domains, all the other conserved domains of *Gossypium* GNAT, CBP and TAF_II_250 were largely similar to their counterparts in monocots and dicots (Figure 2C). These observations suggest that all the *Gossypium* HATs may have similar functions as described in other plant species.

Gene duplication plays a significant role in generating new gene subfamilies in the evolution of genome and genetic systems [41]. Tandem duplication, polyploidy, and segmental duplications primarily contribute to the creation of new gene families [41]. Two segmental duplications were found in *G. hirsutum* (*GhHAC1501A/GhHAC1502A, GhHAG1501A/GhHAG1502A*), *G. raimondii* (*GrHAC1501/GrHAC1502, GrHAC1502/GrHAC1503*), and *G. arboretum* (*GaHAC1501/GaHAC1502, GaHAG1501/GaHAG1502*) (Figure 3). Gene duplications occurred in these genes because the identities of the genes flanking both sides of the paralogous *Gossypium HAT* genes were found to be absolutely conserved and located on duplicated segments on two different chromosomes. Moreover, these duplicated genes were not likely diverged much during the evolution based on their Ka/Ks ratios (Table S2), suggesting the functional conservation of duplicated genes. This observation can partially be validated by the overlapping expression patterns of *GhHAG1501* and *GhHAG1502* during the fiber development and in different tissues (Figure 5A,B). However, the expression of *GhHAG1501* did not change in response to Cd, while the expression of *GhHAG1502* increased compared with control (Figure 6), suggesting that these duplicated genes may undergo functional divergence in response to particular stimuli.

*Arabidopsis* HATs play a crucial role in different aspects of plant development [12,14,15,16,18]. *G. hirsutum* has a huge contribution in the textile industry. Therefore, the identification of *HATs* genes in *G. hirsutum* and their role in fiber development will provide the fundamental information for future studies. Our results showed that the expression of *GhHAC1503* (CBP subgroup) was increased during the different stages of fiber development, while the expression of *GhHAG1501/GhHAG1502* (GNAT subgroup) and *GhHAF1501* (TAF_II_250 subgroup) was generally decreased (Figure 5B). This suggests that histone acetylation levels are likely to be dynamic during the course of fiber development. However, further studies are required to investigate the function of histone acetylation in cotton fiber development. Phytohormones, including gibberellic acid (GA) [42], jasmonic acid (JA) [43], abscisic acid (ABA) [44], ethylene [45], and auxin (NAA) [46], are also known to regulate the fiber development. Cis-regulatory elements specific for ethylene (ERE), GA (GARE), ABA (ABRE), and JA (CGTCA) were found in the promoter of four highly expressed *GhHATs* (*GhHAC1503, GhHAG1501, GhHAG1502* and *GhHAF1501*) during the fiber development (Tables S3 and S4, Figure S3). This suggests that the expression of these *GhHATs* might be regulated by phytohormones. Consistent with this, the expression of *GhHAG1501/GhHAG1502* slightly increased in response to ABA treatment, while the expression of *GhHAC1503* decreased (Figure 6). The expression of *GhHAF1501* did not change in response to ABA treatment, but the expression was increased in response to NAA treatment (Figure 6). This suggests that these phytohormones directly or indirectly regulate the expression of these *GhHATs*, which may lead to different acetylation levels. The exogenous application of auxin has been shown to cause higher acetylation levels at the promoter of *SKP2B* [47], while the acetylation levels decrease at the promoter of *KRP7* in *Arabidopsis* [48], indicating the crosstalk between phytohormones and histone acetylation in plants. However, further studies are required to investigate the crosstalk between histone acetylation and phytohormones in cotton. Together, our results suggest that histone acetylation might play an important role in fiber development, as well as cotton growth. However, further studies are required to investigate the function of each GhHATs in phytohormone related pathways.

Histone acetylation has been reported to play a key role in DNA damage repair [49,50,51]. Histone acetyltransferase 1 (HAT1) is required for the incorporation of H4K5/H4K12 acetylated H3.3 histones at the sites of double strand breaks (DSB), which then facilitate the recruitment of a key DNA repair factor Rad51 in mammals [52]. Furthermore, DSB-inducing agents have been reported to induce the H4K16ac levels in mammals [53], indicating a key role of histone acetylation in DNA damage and repair pathways. In *Arabidopsis*, the histone acetyltransferases HAM1, HAM2 and HAG3 have been reported to participate in UV-B induced DNA damage [54,55]. Furthermore, a treatment with the histone acetyltransferase inhibitor curcumin increased DNA damage in *Arabidopsis* and maize [54], indicating the conserved role of histone acetylation in DNA damage in plants. Our qRT-PCR results showed that the expression of *GhHAC1502, GhHAC1503, GhHAG1501* and *GhHAF1501* increased, while the expression of *GhHAC1504* decreased compared with the control in response to MMS treatment (Figure 6). In brief, our results suggest the potential implication of GhHATs in DNA damage repair pathways in cotton. However, further studies are required to investigate whether and how GhHATs are involved in DNA damage repair.

Different abiotic stresses, including, cold, drought, salt, and metal stresses, severely affect the cotton growth and yield. Recent studies established the link of histone acetylation and abiotic stresses [32,56,57,58]. For example, salt treatment caused a global increase of H3K9 and H3K4 acetylations [32], while cold treatment decreased the H3K9, H4K5 and H4K4 acetylations compared with the control in maize [58]. Similarly, in response to dehydration treatment, drought responsive genes such as *RD29A*, *RD29B*, *RD20* and *RAP2.4* were found to be differentially acetylated at H3K9, H3K14, H3K23 and H3K27 in *A. thaliana* [56]. We showed that many *GhHATs* were differentially regulated in response to different abiotic stresses, suggesting the dynamic levels of histone acetylation in each abiotic stress adaptation in cotton. Our expression data also suggest the functional diversity and specificity among different subgroups of *Gossypium* HATs (GhHACs, GhHAGs and GhHAFs) in response to stress, as well as hormone treatments (Figure 6). For example, the expression of *GhHAC1502* strongly increased in response to NaCl, while the expression of *GhHAC1503* was strongly induced by Zn. Similarly, in the HAGs subgroup, the expression of *GhHAG1501* was strongly induced by NaCl, while the expression of *GhHAG1502* did not change. These observations suggest that GhHATs likely play an important role in cotton plant adaptation to various abiotic stresses. Furthermore, we also noticed that more than one gene responded to a particular stress. For example, in response to salt stress, the expression of *GhHAC1501, GhHAC1502, GhHAC1503, GhHAG1501, GhHAG1504* and *GhHAF1501* increased, while the expression of *GhHAC1504* and *GhHAG1503* decreased compared to the control. This suggests that different GhHATs may work together in response to particular stimuli and may participate in long-term resistance to different abiotic stresses. However, further molecular and biochemical studies are required to validate GhHATs function and to understand the underlying molecular mechanism.

Four *HAT* genes (*GhHAC1502*, *GhHAC1503*, *GhHAG1503* and *GhHAG1504)* on six chromosomes were anchored in fiber development-related QTL regions. These four genes were identified inside multiple QTLs, suggesting the pleiotropic role of these genes in fiber related traits. These results also support the conclusion that both the A and D genomes of *Gossypium hirsutum* jointly participate in different aspects of cotton fiber trait. Interestingly, *GhHAC1502*, *GhHAC1503*, *GhHAG1503* and *GhHAG1504* also displayed differential expression at different fiber development stages and in response to abiotic stresses, metal stress, MMS and phytohormones. This suggests that these four *HAT* genes are potentially involved in cotton growth and development, fiber-related traits, and plant response to the environment. However, in general, there were few discrepancies between RNA-seq and qRT-PCR data. RNA-seq expression levels were either from the A or D genome of *G. hirsutum* and it would be difficult to properly separate and count the reads from the homologous regions. On the contrary, we regarded *GhHACxA* and *GhHACxD* as one combination, referred to as *GhHACx*, because of the extremely high similarity between the mRNAs of the *GhHACxA-GhHACxD* gene pairs (for example, *GhHAC1502A/GhHAC1502D* and *GhHAG1501A/GhHAG1501D* have 99 and 99.5% similarity, respectively) and their nearly identical transcript sizes. With such a high similarity, we could not distinguish them using the qRT-PCR, and the results of qRT-PCR are theoretically the average of A and D genome’s expression. Thus, potential difficulties of read counts on homologous regions in RNA-seq and inability to separate the A and G genome expressions by qRT-PCR may cause the discrepancy between RNA-seq and qRT-PCR results. In brief, further studies are required to comprehensively elucidate the function of HATs in different aspects of cotton plant.

In this study, we highlighted complex and diverse transcriptional regulations of *GhHATs* which significantly broadened our understanding of the underlying epigenetic mechanisms in allotetraploid cotton and unraveled an extra layer of complexity for better allotetraploid cotton adaptation in response to developmental, environmental, and hormonal cues. Furthermore, our results strongly recommend the comprehensive dissection of the biological and cellular function of GhHATS and argue for the potential implication of histone acetyltransferases in cotton molecular breeding in addition to other existing breeding strategies. This will eventually lead to the long-term improvement of stress tolerance in cotton and will allow high fiber yield and quality.

## 4. Materials and Methods

### 4.1. Identification of HATs Gene Family

The data of three cotton species, *G. raimondii* (JGI, version), *G. arboretum* (BJI, version 1.0), and *G. hirsutum* (NAU, version 1.1) were attained from the COTTONGEN (http://www.cottongen.org) [33,34,35]. The HAT protein sequences from *Arabidopsis*, rice, *Brachypodium distachyon*, and *Vitis Vinfera* were downloaded from Phytozome (http://phytozome.jgi.doe.gov/pz/portal.html) and then used as queries in BLASTP searches [59] against the *G. raimondii, G. arboretum* and *G. hirsutum* genomes, respectively. Genes with *E*-values < 1.0 were selected, and redundant sequences were eliminated by following the previously published method [60]. Furthermore, InterProScan (http://www.ebi.ac.uk/interpro/search/sequence-search) was used to confirm the presence of the HAT domain [61]. The physicochemical properties were predicted by ExPASy (http://cn.expasy.org/tools).

### 4.2. Analysis of Chromosomal Location and Gene Duplication

The loci of *HATs* were deduced from the gff3-files of cotton genome. The localization of *HATs* genes on the chromosomes were visualized using the program Circos [62]. The duplication events of *HATs* and Ka/Ks were calculated using the previously published method [63]. The T = Ks/2λ equation was used to determine the duplication time and deviation of the *HAT* gene pairs, assuming clock-like rates of (λ) 1.5 × 10^−8^ substitutions per synonymous site per year for cotton [64].

### 4.3. Sequence Alignment and Phylogenetic Analyses

Multiple sequence alignment was performed for the full-length HAT proteins using Clustal W with standard settings [65]. A neighbor-joining (NJ) phylogenetic tree was constructed using the full length HATs sequences from *G. raimondii, G. arboretum* and *G. hirsutum* by MEGA 6.0 [66], with P-distance and pairwise gap deletion parameters engaged. The bootstrap test was used with 1000 replicates to evaluate the statistical consistency of each node. To confirm the grades from the NJ method, the minimal-evolution method of MEGA 6.0 was utilized with 1000 replicates as well.

### 4.4. Gene Structure, Protein Motif, and Promoter Cis-Element Analysis

The exon/intron structures of the *HAT* genes were acquired from bed-file and displayed using the online tool Gene Structure Display Server (http://gsds.cbi.pku.edu.cn) [67]. The NJ tree was constructed with MEGA 6.0 as explained above. The deduced HAT protein sequences of three cotton species were submitted to the online Multiple Expectation Maximization for Motif Elicitation (MEME) version 4.11.1 (http://meme-suite.org/tools/meme) [68] as described [60]. For cis-element analysis in promoter regions, the 1 kb upstream sequences were analyzed in the PlantCARE database (http://bioinformatics.psb.ugent.be/webtools/plantcare/html/) [69].

### 4.5. Transcriptome Data Analysis and Gene Expression Heatmap

The raw data of RNA-seq of *G. hirsutum* were downloaded from the NCBI Sequence Read Archive (SRA: PRJNA248163) to calculate the expression level. TopHat (version: 2.0.13) was used for mapping reads, cufflinks (version: 2.2.1) were used to analyze gene expression levels, and fragments per kilobase million values were used to normalize gene expression levels [70]. Finally, these values of the *GhHAT* candidates were extracted from total expression data and the heatmap was generated by MeV 4.0 (http://www.tm4.org/).

### 4.6. Plant Materials, Stress Treatments, and qRT-PCR

*G. hirsutum* cultivar ‘CRI35’ was used for gene expression analysis. All the sampled tissues obtained from cotton plants grown under field condition with standardized cultural practices to determine the expression analysis [60]. For treatments, cotton seeds were surface sterilized and germinated on a moist paper. Young seedlings of same size were selected and exposed to NaCl (200 mM), polyethylene glycol 4000 (PEG4000) (15%), cold (4 °C), methyl methanesulfonate (MMS) (250 ppm), auxin (NAA) (10 µM), abscisic acid (ABA) (10 µM), ZnSO_4_ (1 mM), and CdCl_2_ (1 mM) for 24 h. All treatments were performed in three biological replicates. All samples were frozen quickly in liquid nitrogen and kept at −80 °C. The total RNA was extracted from cotton samples using the RNAprep Pure Plant kit (TIANGEN, Beijing, China). A total of 2 μg of RNA was used as the template, and the first-strand cDNAs were synthesized using the SuperScript III (Invitrogen, Waltham, MA, USA). Quantitative real-time PCR (qRT-PCR) analysis was performed as described previously in [71] using the specific primers for each *GhHAT* gene (Table S5). Cotton *UBQ7* (UniProt accession number: AY189972) was used as an internal reference gene for normalization of expression and three biological replicates were performed for each sample. To calculate the relative expression levels, a comparative 2^−∆∆*C*t^ method was used [72]. The heat map for the gene expression profiles was generated with Mev 4.0 (http://www.tm4.org/) [73].

### 4.7. Co-localization of HATs with Fiber Related QTLs

To identify the localization of QTLs and SNPs for fiber development related traits, QTLs and linked molecular markers were retrieved from the Cotton Gen website (https://www.cottongen.org). The sequence of each marker was fetched from Cotton Gen to obtain the physical position information. For this purpose, the sequence of each marker was BLAST against the *G. hirsutum* (AD1) and HAU genome in the CottonGFD database. HAT genes co-localized with QTLs were displayed to show *HAT* gene distribution on chromosomes, along with surrounding loci and QTLs, using mapchart software. Genes identified inside the QTL or ≤500 kb far from SNP were considered as anchored gene in QTL because cotton LD decay was approximately 0.80 Mb [74].

### 4.8. Statistical Analysis

After performing normal distribution of the data and the homogeneity of variance tests, analysis of variance (ANOVA) was performed, followed by the least significant difference (LSD) test at *p* value ≤ 0.05 for each parameter. Different letters indicate significant difference by LSD test (*p* ≤ 0.05). Statistical analyses were performed using the Statistical Package for Social Sciences (SPSS) software (version 11.5, SPSS Inc., Chicago, IL, USA).

## 5. Conclusions

In this study, 36 HATs were identified in three genomes of *Gossypium* and clustered into three groups: CPB, GNAT, and TAF_II_250. *Gossypium HATs* are unevenly distributed on the chromosomes, and segmental duplications contributed to the evolution of the *HATs* family. Cis-element analysis discovered several abiotic and biotic stresses and hormonal responsive elements in the promoter region of the *GhHATs*, but each member had peculiar types and numbers. Furthermore, the expression profile of the upland cotton *HAT* gene family exhibited different expression patterns in response to abiotic and hormonal stresses, which disclosed that GhHATs play roles in different aspects of upland cotton abiotic stress tolerance and hormonal signaling. Thus, our study helps to lay the foundation for the functional characterization of the *GhHATs* gene family by overexpression and knockdown/out using RNAi or CRISPR-Cas9 genome editing and provides new insight into the evolution and divergence of *HAT* genes in plants. Furthermore, these results may enhance the understanding of the molecular mechanisms of many agronomic traits of cotton, such as fiber development and other physiological processes.

## Supplementary Materials

Supplementary Materials can be found at https://www.mdpi.com/1422-0067/20/21/5311/s1.
