# Supplementary figures and images for: Comparative Genome-wide Analysis and Expression Profiling of Histone Acetyltransferase (HAT) Gene Family in Response to Hormonal Applications, Metal and Abiotic Stresses in Cotton
Source: Int J Mol Sci. 2019 Oct 25;20(21):5311. doi: 10.3390/ijms20215311 (PMC6862461; doi:10.3390/ijms20215311)

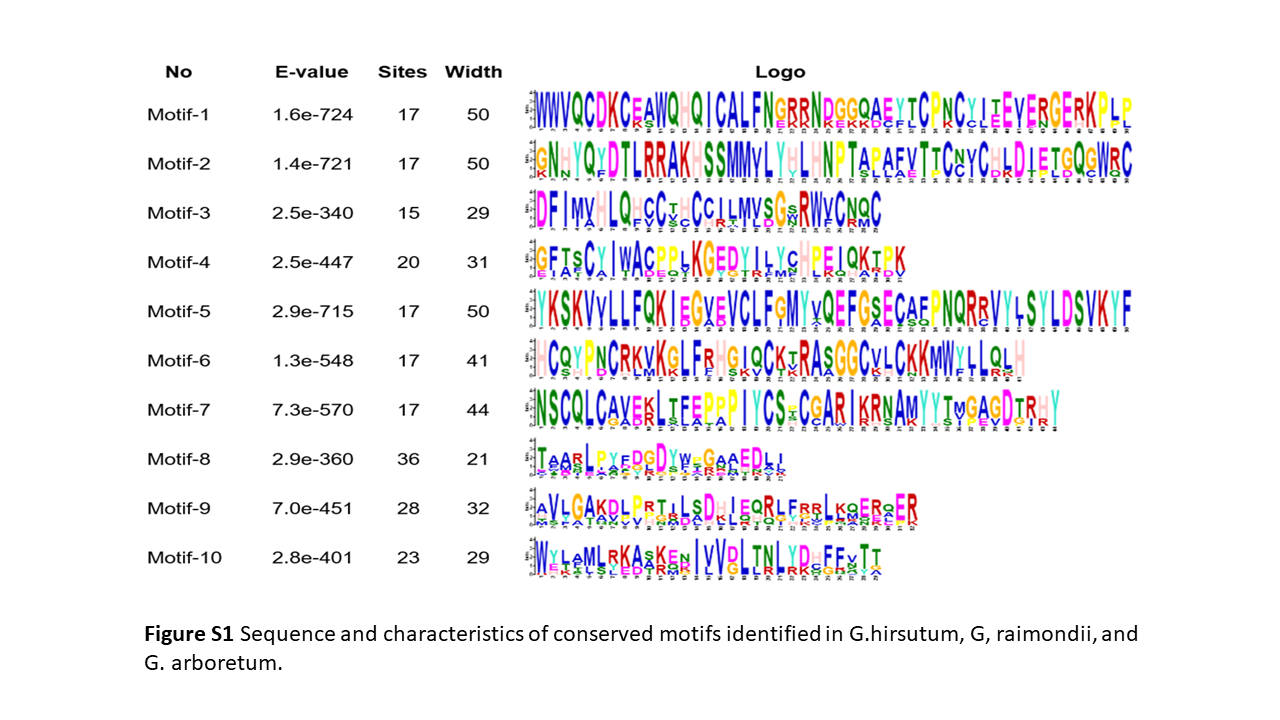

Supplement: Supplementary file 1 [file ijms-20-05311-s001.zip › Figure S1.tif]

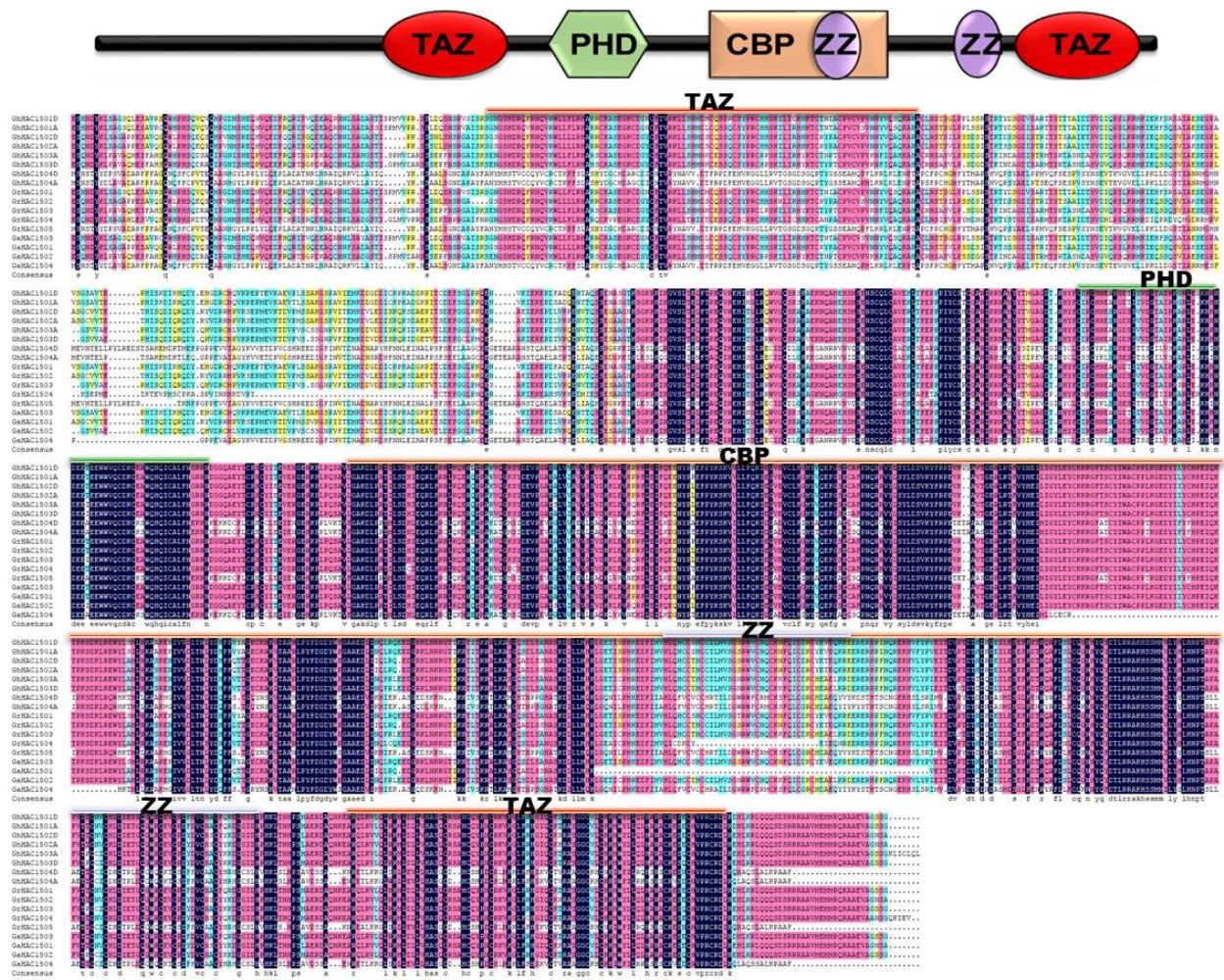

Figure S2 A

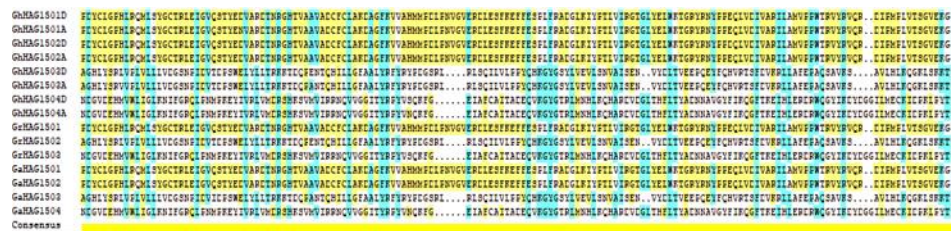

Figure S2 B

Supplement: Supplementary file 1 [file ijms-20-05311-s001.zip › Figure S2.pdf]

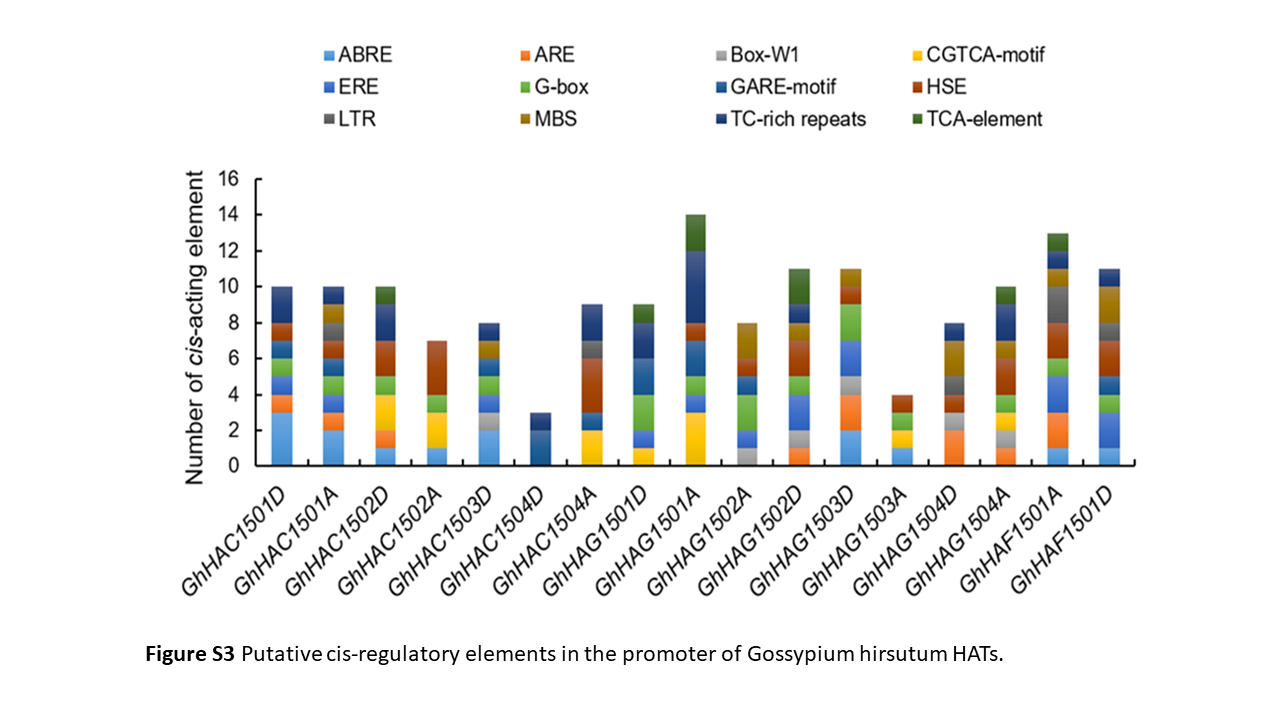

Supplement: Supplementary file 1 [file ijms-20-05311-s001.zip › Figure S3.tif]
